# Seasonal acoustic presence of marine mammals at the South Orkney Islands, Scotia Sea

**DOI:** 10.1098/rsos.230233

**Published:** 2024-01-03

**Authors:** Linn Åsvestad, Heidi Ahonen, Sebastian Menze, Andrew Lowther, Ulf Lindstrøm, Bjørn A. Krafft

**Affiliations:** ^1^ Institute of Marine Research, 5005 Bergen, Norway; ^2^ Norwegian Polar Institute, 9296 Tromsø, Norway; ^3^ University of Tromsø, 9037 Tromsø, Norway; ^4^ Institute of Marine Research, 9296 Tromsø Norway

**Keywords:** marine mammals, species composition, biodiversity, passive acoustic monitoring, vocalization, Scotia Sea

## Abstract

Increased knowledge about marine mammal seasonal distribution and species assemblage from the South Orkney Islands waters is needed for the development of management regulations of the commercial fishery for Antarctic krill (*Euphausia superba*) in this region. Passive acoustic monitoring (PAM) data were collected during the autumn and winter seasons in two consecutive years (2016, 2017), which represented highly contrasting environmental conditions due to the 2016 El Niño event. We explored differences in seasonal patterns in marine mammal acoustic presence between the two years in context of environmental cues and climate variability. Acoustic signals from five baleen whale species, two pinniped species and odontocete species were detected and separated into guilds. Although species diversity remained stable over time, the ice-avoiding and ice-affiliated species dominated before and after the onset of winter, respectively, and thus demonstrating a shift in guild composition related to season. Herein, we provide novel information about local marine mammal species diversity, community structure and residency times in a krill hotspot. Our study also demonstrates the utility of PAM data and its usefulness in providing new insights into the marine mammal habitat use and responses to environmental conditions, which are essential knowledge for the future development of a sustainable fishery management in a changing ecosystem.

## 1. Introduction

The strongly seasonal and highly productive Southern Ocean (SO) supports a high standing stock of Antarctic krill (*Euphausia superba,* hereafter krill) [1,2]. These crustaceans constitute the main prey of a wide range of taxa (e.g. fish, squid, seabirds, pinnipeds and cetaceans), and supports the most diverse and abundant marine mammal community on Earth [3,4]. The Antarctic Peninsula (AP) and Scotia Arc region has been identified as the area with highest concentration of krill within the SO, and as prime target area for both the commercial krill fishery and large aggregations of marine mammals [4–7]. Despite several species of marine mammals almost being eradicated after the commercial harvest during the nineteenth and twentieth centuries and the Scotia Sea being one of the most heavily exploited areas, there have been clear signs of population recovery over recent decades [2,8–13]. Considering ongoing and future climatic changes, and with the AP and Scotia Sea being among the fastest-warming areas on Earth [14,15], there is an increasing need to gain knowledge about the cascading effect that such environmental changes may have on the distribution patterns and ethology of the major krill predators. Additionally, the operation of the commercial krill fishery in this region has the potential to affect the marine mammal consumption requirements by removing or displacing prey and thus affecting their distribution [13,16–18]. For future conservation and management approaches, it is of high priority to increase our understanding and knowledge of local marine mammal community structure and habitat use.

The AP and Scotia Sea region has been recognized as an important feeding area for several species of marine mammals [19–22], a probable result of high prey abundance [4]. The South Orkney Islands, located in the Scotia Sea, have high seasonal phytoplankton productivity and krill density, probably related to the seasonal sea ice cover, nutrients, bathymetric features and inflow from the Weddell Sea [1,23,24]. As a result, the South Orkney Islands have developed to become the most important commercial krill fishing ground in the SO and support both residential and migratory predators [1,19]. While phocid seals (Weddell seal (*Leptonychotes weddellii*), leopard seal (*Hydrurga leptonyx*) and crabeater seal (*Lobodon carcinophagus*)), Antarctic minke whales (*Balaenoptera bonaerensis,* hereafter minke whale), and some odontocetes appear to be year-round residents in the SO [6,21,25,26], most large baleen whale species (e.g. humpback whale (*Megaptera novaeangliae*) and fin whale (*Balaenoptera physalus*)) perform long-distance seasonal migration between their low-latitude winter breeding grounds and high-latitude summer feeding grounds [27]. However, Antarctic blue whales (*Balaenoptera musculus intermedia,* hereafter blue whale) have shown year-round presence in the SO [8,28], and Schall *et al.* [29] provided evidence that some humpback whales may skip southward migration, and some may use alternative feeding grounds in response to low prey abundance. Important drivers behind the timing of the large baleen whales' migration to and from their austral summer feeding grounds are thought to be the seasonal fluctuations in food availability and sea ice extent [20,27,29].

Migration patterns and habitat use of marine predators are also likely to be influenced by changes in environmental conditions, and such trends are especially evident in polar regions where even small temperature fluctuations can result in extensive environmental perturbations [11,30,31]. The El Niño-Southern Oscillation (ENSO) and the Southern Annular Mode (SAM) are two climatic oscillations strongly affecting the SO ecosystems. While ENSO originates in the tropical pacific and affects global atmospheric circulation, thus having pronounced effects on temperature, precipitation and ocean currents, SAM is the leading pattern of natural climate variability in the Southern Hemisphere [32–36]**.** The strength of the ENSO teleconnection to the Southern Hemisphere depends on the coupling with SAM. When the two are ‘in phase’, meaning that an El Niño (La Niña) event is coupled with a negative (positive) SAM, the ENSO teleconnection to the SO is stronger than average [36]. Though both oscillations have shown to strongly influence the productivity and species assemblage in the SO by affecting sea surface temperature and sea ice dynamics [29,33,35,37], the direct effect of such climatic changes at high-latitude regions is still poorly understood. In areas such as South Orkney Islands, which is characterized by ecosystem variability, it is of both commercial and ecological importance to be able to predict inter-annual changes in krill distribution and density. Presumably, the South Orkney Islands do not have a self-sustaining krill population [38]. Instead, the island region is thought to act as a sink retaining krill advected from spawning grounds in the AP and Weddell Sea [24,39,40]. Reduced krill survival in response to extreme weather events and changing environmental conditions in these important spawning grounds could reduce the number of krill reaching the South Orkney Islands. Changes in prey availability will subsequently affect the distribution and survival of the upper-trophic level predators [11,37,41].

Attaining population level information on marine mammals' habitat use in time and space in general is challenging and even more so in the SO due to its remoteness and the logistical effort required. To date, vessel- and aerial-based surveys during the austral summer months have been the primary means by which to study marine mammal distribution patterns [42–45]. However, these surveys rely on complex logistic operations with high economic costs [46,47] and are less suitable to assess diversity, distribution and abundance during the remaining annual period.

Over the last two decades, passive acoustic monitoring (henceforth, PAM) has become an important tool in monitoring year-round distribution and habitat use of marine mammals and, as a result, quantifying ecological interactions and how predators respond to anthropogenic pressure [43,48]. For marine mammals, sound is the primary mode used for communication and social interactions, as well as navigation and foraging [25,49,50]. Visual cues are less useful for marine organisms, especially in polar regions during winter, due to the poor underwater transmission of light [49,51]. However, sound propagates much more efficiently and over longer distances [6,52], and some species of marine mammals have evolved a unique vocal repertoire with different degrees of diversity and complexity. The vocal repertoire of a species can be determined by both sex and sexual maturity, and the degree of vocalization may vary between seasons [25,53–55]. For example, while humpback whales produce many different types of social calls, only male humpbacks are known to produce highly complex, long-lasting songs associated with breeding and migration [54,56,57]. By contrast, male blue whales and fin whales produce simple low-frequency calls [2,22,52]. As a result of such well-documented species-specific vocalizations and the underwater propagation properties of sound, PAM technology has demonstrated to be one of the most cost-effective methods to sample long-term data in remote areas.

In our study, we used 16 months of acoustic recordings, spanning over the years 2016 and 2017, from a known krill hotspot at South Orkney Islands to identify the acoustic presence of marine mammals. We identified species-specific vocalizations to gain insight into species diversity and compared trends in species composition between and within years and seasons. Additionally, the association between variability in environmental conditions connected to the strong El Niño event throughout 2016, and the acoustic phenology were explored through multivariate statistical analysis and visualizations.

## 2. Material and methods

### 2.1. Study location and passive acoustic data collection

**Figure 1.**
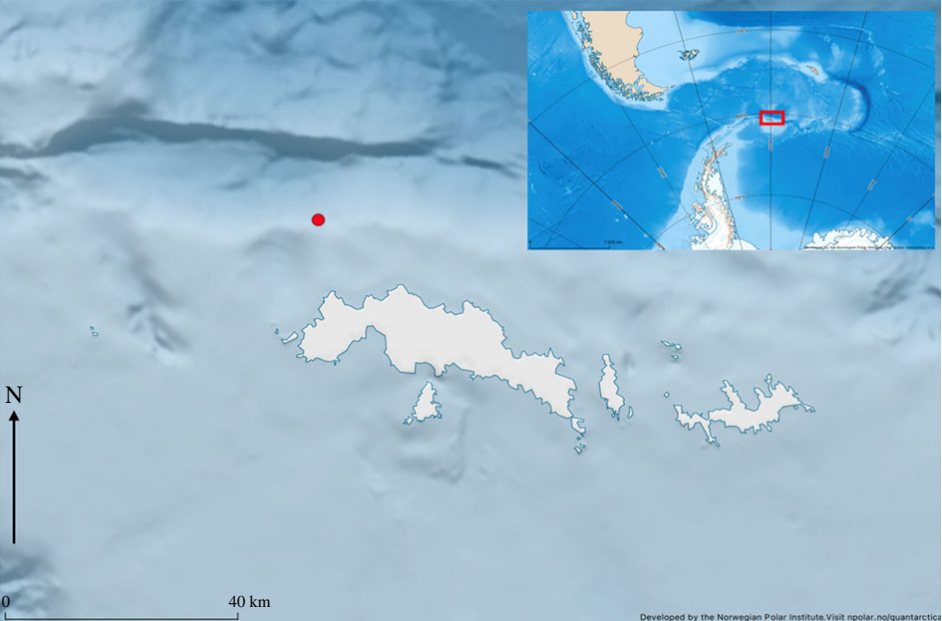
Study area. The South Orkney Islands, located at the southern edge of the Scotia Sea. The red dot indicates the location of the AURAL (Autonomous Underwater Recorder for Acoustic Listening) in the Coronation Trough to the west of Coronation Island, used for the collection of passive acoustic data during 2016 and 2017.

The South Orkney Islands are located approximately 600 km northeast of the tip of the AP, Scotia Sea (figure 1). We collected PAM data over a two-year period using an Autonomous Underwater Recorder for Acoustic Listening (AURAL M2, Multi-Électronique Inc.; receiving sensitivity: −165 dBV µPa^−1^) as part of ongoing ecosystem monitoring around the South Orkney Islands [1,58]. Full deployment details for each year are presented in table 1. The AURAL was deployed on a mooring anchored to the sea floor in the Coronation Trough northwest of Coronation Island. In 2016, the AURAL recorded data between February and August with an hourly duty cycle of 12 min (12 min ON, 48 min OFF). To prolong the recording period, the AURAL settings were adjusted before it was subsequently redeployed in February 2017, where it recorded data from February to October, with an 8 min duty cycle (8 min ON, 52 min OFF). The AURAL's maximum sampling rate (32 768 Hz) excluded the possibility to record high-frequency vocalization of most odontocete species, but sufficiently covered the range in which baleen whales and pinnipeds vocalize.

**Table 1.** Deployment details. Deployment details for the AURAL M2 (Autonomous Underwater Recorder for Acoustic Listening), with HTI-96-min hydrophone, used in 2016 and 2017 at South Orkney Islands.

year	recording period	coordinates	bottom depth/ recorder depth (m)	duty cycle/cycle time (min)	sampling rate (Hz)	frequency response range (Hz)^a^	gain (dB)
2016	16 February–23 August	60°24.297' S, 045°57.548' W	470/240	12/60	32 768	2–30 000	16
2017	10 February–12 October	60°24.281' S, 045°58.311' W	479/286	8/60	32 768	2–30 000	16

^a^Hydrophone specifications provided by manufacturer High Tech Inc (http://www.hightechincusa.com/products/hydrophones/hti96min.html).

### 2.2. Passive acoustic data analysis

All acoustic recordings used for call identification were screened as spectrograms and manually annotated using Ishmael BioAcoustics (v. 3.0.2) with fixed parameters (Hann window, Hop size: 0.5, frame size (FFT): 4096, no zero padding). To evaluate whether the difference in duty cycle between the two years affected call detection, the 2016 acoustic recordings were annotated with an 8 min mark. Resultingly, we could see to what degree species appeared solely after the 8 min. The difference in duty cycle appeared to not affect call detection, and as such, we used the full 12 min of recording in our analysis. The target species for this study were all potential species that might reside in the area, but with specific interest in krill predators (baleen whales and pinnipeds). Erbe *et al.* [59] presents an overview of species that have been observed south of 60°. However, with krill predators being the main focus in our study, all spectrograms were visualized with a window of 60 s duration and a constant frequency range of 0–8 kHz as the vocalization of these species can be found within this same frequency range [2,25,29,60,61]. Python Audio Spectrogram Explorer (PASE; [62]) was run simultaneously to Ishmael, looking at the same acoustic file but concentrating solely on the low frequencies (PASE settings: frequency range: 0–110 Hz, linear scale, FFT: 32789, FFT overlap: 0.9, spectrogram length: 120 s) to annotate blue whale and fin whale calls. These low frequency calls would be difficult to detect from spectrograms with frequency range 0–8 kHz. Potential calls observed in the spectrograms were inspected aurally to distinguish between marine mammal calls and sounds of abiotic origin. Time and frequency range were adjusted during closer inspection of vocalizations, after which they were classified into species where possible. Subsequently, the species were categorized after their ice-affiliated or ice-avoiding nature. Identification of species-specific vocalizations were based on previous work (e.g. [25,60,63–65]). Due to krill predators being our main focus, and species identification and manual annotation is a time-consuming process, odontocete vocalizations (clicks, whistles and burst pulses) detected within the set spectrogram range were annotated and included as one group rather than differentiated between species, to show that also this group of cetaceans are present in the area. When encountering vocalizations of low certainty, these were cross-checked with other analysts, and in the case of not being able to confidently identify the species, the detection was removed from further analysis. Note that though different call types were detected, these were not differentiated in the manual detection log, and species’ presence was based on all call types. The continuous chorus band produced by distant blue whales and fin whales found between 18 and 28 Hz [2,66] was excluded from this study due to the focus being local habitat use. Each species' acoustic presence was assessed on an hourly basis and was logged if at least one clear and recognizable species-specific vocalization was present in the recording. Our study and results are qualitative only, and thus, regardless of number of vocalizations in one file and the potential for signals coming from several animals, all positive detections were treated as presence-only data. The resulting binary absence/presence data per hour were used to explore time series of daily acoustic presence (percentage of hours per day containing at least one species-specific vocalization).

### 2.3. Estimated audible area

Variability in the detected marine mammal vocal activity cannot only be caused by changes in animal abundance and behaviour, but also by the temporal variability of the area from which sound could be detected by the recorder (here termed the audible area). The extent of this area depends on local noise levels and the transmission loss (TL) between sources and the recorder. The TL, and resultingly propagation distance, is affected by bathymetry (static) and temperature, salinity and sea ice (variable) [48,67–69]. Sea ice cover and surface roughness differ depending on the sea ice age, season and area, and will thus reflect/scatter sound in different ways and resultingly affect the sound propagation [59,70,71]. The growing ice cover as winter progresses can greatly increase the detection range by reducing the underwater noise level, but also reduce detection range due to increased surface roughness. When the surface roughness increase, sound will be increasingly scattered rather than reflected as it would be with a smoother surface [69]. However, low-frequency signals travel further due to their reduced attenuation in seawater and are less affected by sea ice roughness [70]. Free-ice, icequakes and iceberg tremor sounds are common in the ambient soundscape in the Scotia Sea, especially during summer/early autumn. These acoustic signals may mask the biotic part of the soundscape, especially in the lower frequency range (less than 100 Hz) [72]. Additionally, features of the bathymetry can act as physical barriers and cast acoustic shadows or reduce the detection range of calls [69,73]. We addressed these issues by comparing time series of acoustic detections with variability in the audible area. To examine the general temporal pattern in the audible area, the sound propagation model was run for two frequencies (50 and 500 Hz; electronic supplementary material, figure S1) to account for species vocalizing in different frequency ranges. For the statistical analysis, species-specific frequencies and typical source levels for each species used in the GAMM modelling (table 2) were used in the model to make daily estimates of the audible area. These were converted into shapefiles used for extraction of the environmental covariates within the AURAL's range.

**Table 2.** Audible area estimation. Frequency and source level used in modelling estimated audible area for fin whale, Antarctic blue whale, Antarctic minke whale and humpback whale used for extracting spatially restricted environmental variables. Choice of source level was based on literature as well as noise levels in the acoustic recordings. Frequency was based on a combination of literature and visualization of the acoustic recordings.

species	frequency (Hz)	source level (dB re 1 µPa)	reference
fin whale	20	189	Miller *et al.* [74]
Antarctic blue whale	20	189	Miller *et al.* [74]
Antarctic minke whale	100	163	Risch *et al.* [63,75,76]
humpback whale	200	163	Au *et al.* [77]

We modelled the audible area around the recorder using a sound propagation model and reanalysis data [78]. The two-dimensional ray-tracing software BELLHOP was used to model TL, by rotating slices (500 km long) in 1° intervals around the recorder location. For each slice, the bathymetry profile was derived from the GEBCO dataset [79] and temperature and salinity fields (to calculate the sound speed field) as monthly snapshots from the Copernicus marine services ocean-reanalysis dataset [80]. Source depth was set to 15 m and receiver depth to 280 m, bottom density to 1200 g m^−^^2^, bottom sound speed to 1450 m s^−1^ and bottom absorption to 1 dB wavelength^−1^. After calculating the TL profile for each slice, we used linear interpolation to grid the TL values onto a latitude–longitude grid with a mesh size of 464 × 218. Grids of signal-to-noise ratio were then calculated by subtracting the modelled TL and measured noise levels for each recording (and each frequency) from typical source levels for each species. Local noise was calculated as Welch's power spectrum (Hamming window, FFT size 65536) from the first 8 min of each recording. The audible area for each recording was then calculated as the area where the signal-to-noise ratio was larger than 5 dB. These estimates were then averaged over time to provide daily masks of the audible area for each frequency.

### 2.4. Environmental covariates

Bathymetry data with a 0.5 × 0.5 km resolution was provided by the GEBCO Compilation Group [81]. Daily sea surface height anomaly (SSH) with a 27.75 × 27.75 km resolution was provided by Copernicus Climate Change Service information [82]. Daily estimates of global sea surface temperature (SST) with a 5.55 × 5.55 km resolution were provided by UK Met Office [83]. Sea ice concentration (SIC) was obtained from satellite data (3.125 × 3.125 km resolution) processed and provided by the University of Bremen [84]. Time series of daily average SST, SSH, and SIC were plotted to assess potential inter-annual differences (electronic supplementary material, figures S4–S6) and for comparison with species’ acoustic presence. As some marine mammal vocalizations (e.g. blue whale Z-calls) can travel greater than 100 km, while other species' vocalizations only travel a few kilometres [52,85,86], SIC values for a 25 and 100 km radius around the AURAL were calculated. However, comparisons between the two radii were similar, and thus SIC for the 25 km radius was chosen for further analysis.

For the statistical analysis, we spatially constrained daily values for all covariates (bathymetry, SST, SSH, SIC) to the audible area by using the *raster* [87] and *stars* [88] packages in R (v. 4.0.5; [89]). Because the audible area was calculated for four frequencies, the following steps were done four times: (i) The daily proportion of SIC (0–1), with a threshold of minimum SIC greater than 30%, was extracted by overlaying shapefiles of the audible area over each daily sea ice raster and calculating the proportion of area covered by sea ice. (ii) Further, it was assumed that vocalizing animals used in the statistical analysis were constrained by sea ice and that all detections would hence most likely originate from animals within the audible area but outside heavy ice-covered regions or near the ice edge. Therefore, the bathymetry, daily SST and SSH data were converted into rasters and subsequently overlaid by the corresponding daily shapefile of sea ice and audible area to extract their spatially constrained values. All values within the area covered by sea ice were annotated as null values (NA), and subsequently, all values outside the audible area were ignored as this determined the bounding box of the final grid. (iii) Finally, the daily mean SST and SSH and daily coefficient of variation in bathymetry within the audible area were extracted. Though bathymetry is a constant, the variance in bathymetry (vBAT) around the South Orkney Islands is considerable, with a large shelf area, canyon intrusions, and a deep pelagic basin to the north. Thus, the vBAT will decrease as the winter advance of sea ice northwards renders the shelf area less accessible for most species and leaves the relative homogeneous depths of the deep ocean basin to the north available.

### 2.5. Statistical analysis

A constrained correspondence analysis (CCA) was performed for each year to explore large-scale patterns in species assemblage of marine mammals. CCA is a well-suited multivariate method to examine relationships between species and environmental variables and aims to explain shifts or changes in species composition. Though this method assumes linearity between the response (species’ abundance) and predictors (environmental covariates), it approximates unimodal relationships between the two [90,91]. The response was daily acoustic presence (0–24) of each species and month, mean SST, mean SSH, vBAT, and proportion of SIC (0–1) as predictors. The CCA was performed in R (v. 4.0.5; [89]) using the *vegan* package [92].

To examine the impact of SST, SSH, SIC and vBAT on the acoustic presence of marine mammals, Generalized additive mixed models (GAMMs) were fitted using the *gamm* function of the *mgcv* package [93] in R. This model allows for nonlinear relationships between predictor variables [94]. Before fitting the model, predictor variables were scaled and checked for collinearity through the variance inflation factors using the *vif* function from the *car* package [95]. Low values (approx. 1) indicate weak or no correlation, values around 5 indicate moderate correlation, and values greater than 10 indicate strong correlation. All values were between 1 and 3, and all predictors were kept. Quasi-binomial GAMMs were applied to model the daily acoustic presence of blue whale, fin whale, humpback whale and minke whales for each year as a function of SST, SSH, SIC and vBAT. The response variable was daily proportional presence (0–1) of the respective species. Crabeater seal, leopard seal and southern right whale (*Eubalaena australis*) were excluded from the GAMM analysis due to low acoustic presence, and odontocetes were excluded due to the possibility of comprising several species. Though the detection rate for minke whale in 2017 was low, these were included in the analysis for comparative reasons. As the two years presented huge environmental differences, the models were run for each year separately rather than including year as a random effect. Month, however, was added to account for intra-annual seasonal variations. All variables were included as thin plate splines. Smoothness degree (k) was determined as part of the model fitting process and through the restricted maximum-likelihood method (REML). The difference in duty cycle between the two years appeared to not affect call detection and was not included in the model. A *corARMA* term from the *nlme* package [96] was used to account for potential autocorrelation, using the *auto.arima* function (package forecast; [97]) to estimate the order of correlation structure. However, no autocorrelation term was included in the final model. Models were checked for overdispersion, and model evaluation and selection were based on residual analysis through the *gam.check* function, Akaike information criterion (AIC) and adjusted r-squared. Only predictor variables improving the model fit were included in the final model.

## 3. Results

**Figure 2.**
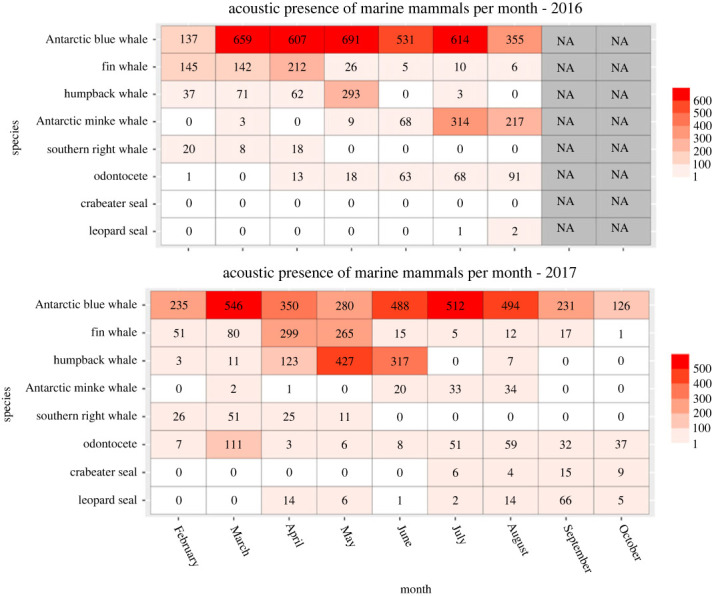
Overall acoustic presence. Heatmap of marine mammal species' acoustic presence by month for 2016 (top) and 2017 (bottom) off the South Orkney Islands. Colour intensity indicates the number of hours per month containing at least one clear detection of the respective species. In 2016 there were no recordings for September nor October due to battery depletion (indicated by NA). Note the different colour scale for 2016 and 2017.

The acoustic recordings revealed seven different species of marine mammals to be present in the area around South Orkney Islands: five baleen whale species (fin whale, blue whale, minke whale, southern right whale and humpback whale) and two pinniped species (leopard seal and crabeater seal). Additionally, odontocete vocalizations constituted an eighth category, though potentially containing multiple species. The overall acoustic presence of each species per month is presented in figure 2. Except for virtually no pinnipeds detected in 2016, both years showed similar seasonal changes in species assemblage. However, peaks, fluctuations and the overall phenology of species' acoustic presence showed inter-annual variations. When excluding blue whale presence due to high vocal activity during all recorded months, there was a clear seasonal variation in overall vocal activity in 2017, and less so in 2016. In 2017, there was a steady increase in vocal activity from February to May, where vocal activity peaked, followed by a decrease from May to October. Though 2016 also displayed an increasing trend in the first part of the year, the overall vocal activity between months was noticeably lower and less variable (min: 136 h, max: 396 h) than in 2017 (min: 52 h, max: 715 h). When referring to austral summer, autumn, winter and spring in the following sections, we are referring to the time periods December–February, March–April, May–September and October–November, respectively.

### 3.1. Temporal trends in species acoustic presence

**Figure 3.**
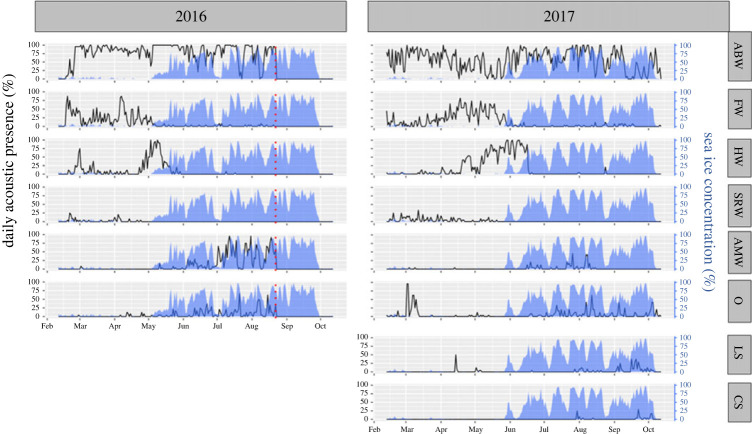
Acoustic presence time series. Daily acoustic presence of marine mammals in 2016 (left) and 2017 (right) recorded by an autonomous underwater recorder located on a mooring northwest of South Orkney Islands. Daily acoustic presence refers to the percentage of hours per day containing at least one species-specific vocalization. Note that there were no recordings from September and October in 2016 (last recording date in 2016 are represented by the red dotted line), and the two months were added for visual purposes. The secondary y-axis and blue shaded area represents daily average sea ice concentration (%) at a 25 km radius around the recorder. The species abbreviations on the right-hand side are ABW = Antarctic blue whale, FW = fin whale, HW = humpback whale, SRW = southern right whale, AMW = Antarctic minke whale, O = odontocetes, LS = leopard seal, CS = crabeater seal.

Time series depicting all detected species’ acoustic presence throughout the recording period can be seen in figure 3, and figures showing detected species-specific vocalizations used for species identification with complementary sound files can be found in the electronic supplementary material, figures S7–S14. Blue whale vocalizations were present throughout both recording periods and were the most frequently detected species. Two major drops in their acoustic presence were detected in July and early August 2016. These periods were separated by a period with high vocal activity, and the drops corresponded well with sudden reductions in the AURAL's audible area (electronic supplementary material, figure S2). Similarly, the oscillating acoustic presence in 2017 presented an overlapping pattern with changes in the audible area during austral winter/spring. Fin whale acoustic presence presented a strong seasonality in both years, with vocalizations virtually disappearing in the onset of austral winter. Inter-annual variations were seen when looking at the peaks. While 2017 presented a gradual increase towards the peak in April before declining, 2016 presented a bi-modal pattern with two peaks. Both humpback whale and southern right whale presented similar seasonality in their acoustic presence to that of fin whales. Humpback whales were detected in late austral summer/early austral winter. Except for a secondary peak in February 2016, both years were dominated by a relatively low number of sporadic vocalizations up until late April, after which vocal activity increased until it peaked in the onset of austral winter. Southern right whale was the baleen whale species with fewest detected vocalizations, and daily acoustic presence remained relatively low both years.

In contrast to the large baleen whales, minke whale vocalizations appeared mainly after the onset of austral winter, and the vocal activity was noticeably lower in 2017 compared with 2016. Additionally, three peaks in 2016, on 12 July, 31 July and 18 August, stood out and were separated by periods with few detections, which coincided with reduction in the audible area (electronic supplementary material, figure S3). Odontocete vocalizations were detected during austral winter/spring, in addition to a peak in March 2017. Crabeater seals presented the lowest acoustic presence of all detected species, with no calls detected in 2016, and only 15 days of 2017 containing calls. Vocalizations were detected in the transition from July–August and September–October, separated by a period of no detections. Leopard seal vocalizations were detected on 25 July and 4 August in 2016, and in 2017 their acoustic presence was higher, though still low, mainly being present from late July to early October.

### 3.2. Environmental correlates with marine mammal acoustic presence

**Figure 4.**
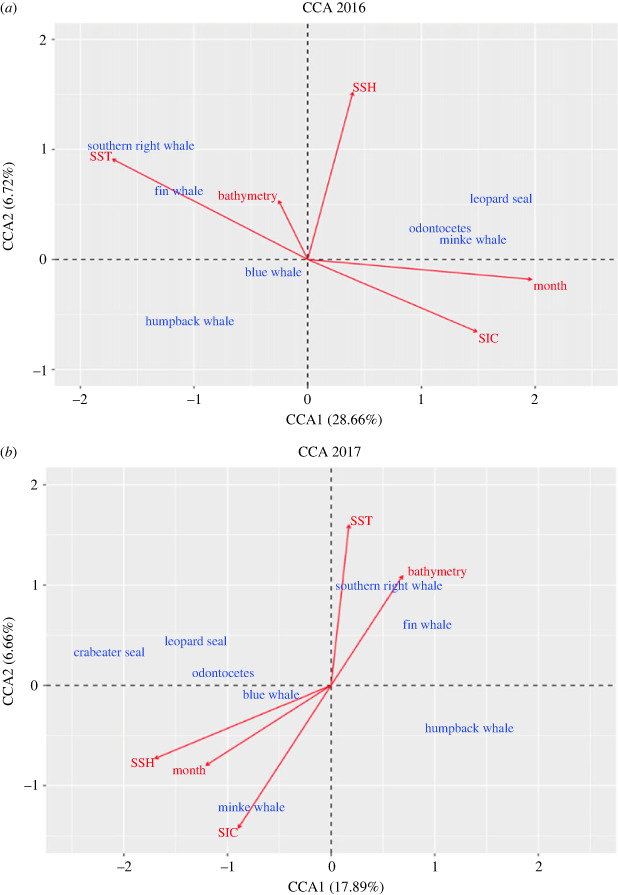
CCA. Constrained correspondence analysis (CCA) biplot relating marine mammal species assemblage to environmental covariates in 2016 (*a*) and 2017 (*b*). The percentage variation in species assemblage explained by CCA axis 1 and 2 is indicated in parentheses in the axis label. Environmental covariates used in the CCA are represented by vectors (red arrows). The labels presented in the figure represent daily mean sea surface temperature (SST), daily mean sea surface height anomaly (SSH), daily proportion (0–1) of sea ice concentration (SIC), variance in bathymetry and month of the year.

A clear seasonal change in species assemblage was observed moving from austral summer/autumn towards winter, separated along CCA axis 1, indicated by the month vector (figure 4). SST and SIC showed to be the leading environmental drivers behind the seasonal change in species assemblage, followed by SSH and vBAT (non-significant in 2016, *p* > 0.05). Southern right whale, fin whale, and humpback whale represent a cluster more associated with increasing SST and variance in bathymetry. Blue whale is centred in the middle without any apparent strong relation to the environmental variables, reflecting their high acoustic presence during the entire recording period. Minke whale, odontocetes, leopard seal and crabeater seal represent a second cluster, associated with increasing sea ice and SSH.

**Figure 5.**
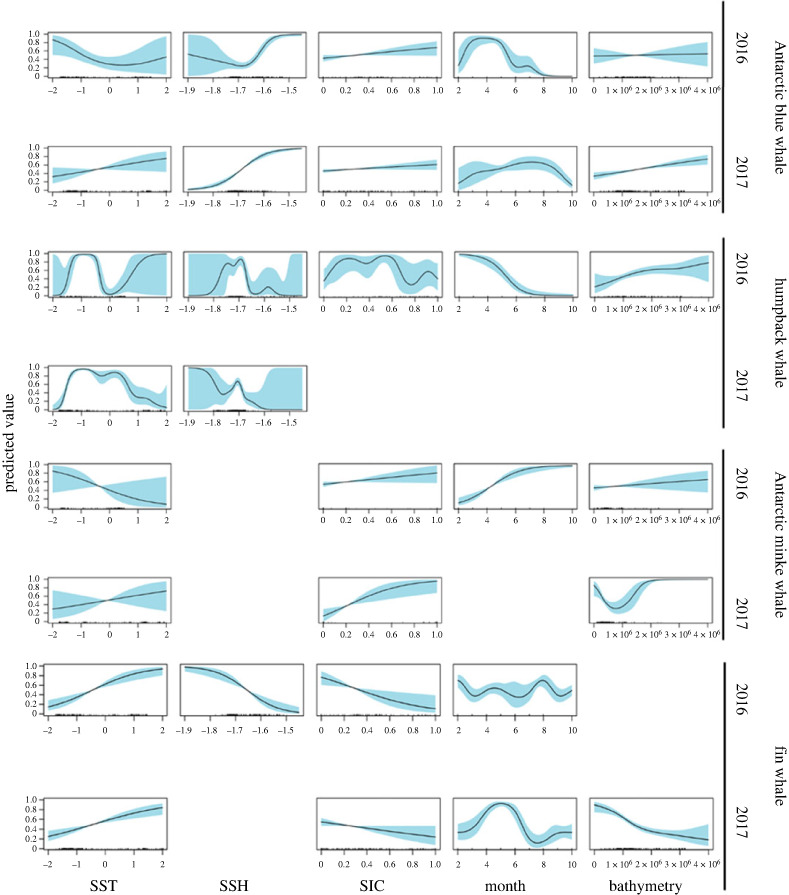
GAMM splines. Splines of generalized additive mixed modelling (GAMM) showing the effect of environmental covariates on acoustic presence of; Antarctic blue whale, humpback whale, Antarctic minke whale, and fin whale in 2016 (n = 188) and 2017 (n = 245). Observations are presented as tick marks on the x-axis, black lines are the estimated splines, and blue-shaded areas represent the 95% confidence intervals. The y-axis shows the effect of each environmental covariate included in the best-fitted model on the probability of acoustic presence, where a low (high) predicted value indicates a reduction (increase) in the probability of acoustic presence. X-axis labels; SIC: sea ice concentration (0–1), SST: sea surface temperature (C), SSH: sea surface height anomaly (m), Bathymetry: coefficient of variance in bathymetry. Missing splines indicate variables not included in the final model. Note that all x-axis scales per covariate is fixed for comparative and visual reasons.

Humpback whale, fin whale, blue whale and minke whale presence varied in relation to the covariates in each year (*p*-values < 0.05). SSH and SST presented a strong significant effect on humpback whale acoustic presence in both years, while in 2016, SIC, vBAT and month also showed a significant effect (figure 5). SIC < 50%, increasing vBAT and low SSH were related to higher humpback whale acoustic presence. Their acoustic presence showed to be highest at SSTs between approximately −1 and 0°C. SST, SIC and month showed a strong significant effect on fin whale presence in both years, while SSH and vBAT were significant in 2016 and 2017, respectively (figure 5). Fin whale presence presented strong seasonal variation, declined with increasing SIC and increased with increasing SST. SIC and month showed to be the most important predictors behind minke whale acoustic presence, followed by SST and variance in bathymetry. Their acoustic presence presented strong seasonal variation, and increased in tandem with increasing SIC. All predictors except SIC and SST showed a strong significant effect on the acoustic presence of blue whale in 2017, while in 2016 only SSH and month were highly significant.

## 4. Discussion

This is the first study to investigate the temporal variation in marine mammal species occurrence and assemblage at South Orkney Islands. We show that (i) there was little seasonal variation in species richness, and (ii) there was an apparent seasonal variation in species assemblage. Note that odontocete vocalizations were treated as one group and may comprise several species. The seasonal variation in environmental conditions segregated the detected species into two clear guilds (ice-avoiding and ice-affiliated), and the inter-annual variability in environmental condition, reflecting the El Niño event, had an apparent effect on the marine mammals' acoustic phenology.

The marine mammal acoustic data allowed for exploration of species assemblage, movement and acoustic phenology, which is important for predicting future responses to changing environmental conditions. The latter is especially relevant for this study as our acoustic data came from two years representing contrasting physical conditions due to the strong 2016 El Niño event [98]. The year 2016 was characterized by noticeably higher SSH values during late summer/autumn compared with 2017 and abnormally low spring sea ice due to increased SST (electronic supplementary material, figures S4–S6). The latter continued to affect the summer/autumn season in 2017, though both the sea ice extent and SST showed little inter-annual variation by mid-winter. Despite the AURAL's battery depletion during summer months limiting the exploration of fully year-round patterns, our study presents valuable information for future research and increases our knowledge about marine mammals’ habitat use at the South Orkney Islands. The high seasonal and inter-annual differences in environmental characteristics at South Orkney Islands during the recording period provided a strong foundation for comparing species distribution and assessing the effect of a changing environment on their acoustic phenology.

### 4.1. Species occurrence and temporal variation

Many odontocetes produce sounds with a much higher frequency [99] than the AURAL M2 frequency range (16 kHz) used in our study and, hence, identification of such sounds was probably not within the sensors' range. Southern elephant seals and Antarctic fur seals vocalize in air [100] and were not expected to be detected. The lack of detections of both the Weddell and Ross seals was surprising considering their aquatic mating system and known underwater vocalizations [101]. However, Weddell seals are known to primarily stay closer to the Antarctic coast and breed on the more stable fast-ice during spring [26,102,103]. Ross seals make long trips north of the pack-ice into the pelagic areas of the SO to feed for most of the year and only return to the pack-ice for short periods during summer to breed and moult [103,104]. The acoustic absence of the remaining baleen whale species that might reside in this region (e.g. sei whale (*Balaenoptera borealis*) and pygmy blue whale (*Balaenoptera musculus brevicauda*)) may be due to their distribution not extending as far south as the position of the AURAL [59,105–109].

Similar to most large baleen whale species, blue whales display seasonal migration between lower latitudes and Antarctic waters [66,110]. In contrast to humpbacks, which are known to perform extensive breeding migration to warmer waters during winter [27], little is known about the annual migration dynamics of blue whales. The fact that blue whales were present throughout the recording period in our study not only confirms previous studies showing year-round presence in highly productive regions [64,110], but also suggests that blue whales are partial and/or differential migrants due to life-history and reproductive status [66,111,112]. It should be pointed out that detections made during winter months consisted solely of the Z-calls’ A-unit (electronic supplementary material, figure S9), which has shown to be the strongest part of the call. Shabangu *et al*. [2] reported that such single-unit detection indicates animals vocalizing at a greater distance from the AURAL. As such, though calls were still detectable, the animals may have moved further away from the South Orkney Islands during winter, presumably towards low-latitude breeding grounds or other overwintering areas. Further support comes from the absence of D-calls after the onset of winter, which are thought to function in short-range communication and in relation to foraging [2,113], and that drops in their acoustic presence coincided with reduction in the audible area.

Humpback whales, fin whales and southern right whales (hereafter referred to as ice-avoiding guild) are known to migrate from lower-latitude breeding grounds to Antarctic waters during spring/early summer to feed on large aggregations of krill [22,29,41]. With the two recording periods having such different environmental characteristics, one would expect inter-annual differences in the animals' acoustic presence as a response. However, predicting such patterns is hard both due to knowledge gaps in current research and because the effect of ENSO is highly sector-specific in the SO, and how animals respond will thus depend on the area of interest. While Schall *et al*. [29] detected virtually no humpback vocalizations during the 2016 El Niño year in the Atlantic sector of the SO, we found high acoustic presence at the South Orkney Islands in the same year, though we did find inter-annual differences between the two recording periods. The secondary peak in humpback and fin whale acoustic presence in February/March 2016 may indicate animals moving out of the audible area to locate prey elsewhere due to unsuitable environmental conditions or insufficient krill availability at the South Orkney Islands, before returning at a later time. With krill being presumably transported to the South Orkney Islands through the Antarctic Circumpolar Current (ACC) from spawning and nursery grounds at the Western Antarctic Peninsula, changing environmental conditions in these key habitats may have resulted in less krill being recruited from the peninsula to the island region. Dalla Rosa *et al.* [114] reported that as a probable result of low local prey density, humpback whales travelled between different feeding grounds with a relatively short residency time. Our hypothesis is also supported by the behaviour of another predator for which ‘perfect’ foraging information is known: the commercial krill fishery. In 2016, the krill fishery caught only half of the catch in 2017 in the South Orkney area [115,116] over the same time period (December–April), which might suggest that the abundance of krill in this region was lower in 2016. This may reflect differences in factors such as fishing effort, vessel size and deployment duration, but due to the 2016 El Niño event, we hypothesize that the environmental anomalies following El Niño could have led to reduced krill density in the area. Of course, it is noteworthy to mention that absence of vocal detections is not always synonymous with the absence of animals, as they may simply be silent or becoming harder to detect. The degree of vocal behaviour of marine mammals differs between species and sex. Males often have a higher probability of being detected as these individuals sing in contrast to females, and while some species vocalize year-round, others only vocalize in relation to breeding behaviour [117–119]. PAM data alone can only yield acoustic presence data, and how representative acoustic presence is over physical presence is highly dependent on the likelihood of an animal vocalizing. Resultingly, our study constitutes presence-only as true absence cannot be certainly stated from acoustic data.

The trade-offs between resource acquisition and predation risk plays an important role shaping the behaviour and movement of animals [120]. Given that whales are top or apex predators and have few natural predators, food availability is probably the single most important factor shaping the movement of these marine mammals [121,122]. Sea ice constrains their movements both directly, as a barrier, and indirectly, through changes in prey availability. The cessation of virtually all detectable vocal activity of the ice-avoiding guild happened before the main sea ice formation began in both years, though humpbacks presented some overlap. This reflects their common disassociation with sea ice, and the hypothesis that their distribution follows the position of the ice edge was further evidenced by the observed inter-annual difference in their acoustic phenology. Following an Antarctic-wide decrease of sea ice in austral spring 2016, anomalously high SSTs were observed in large parts of the SO [98], coinciding with observations in the current study. The mean SST in the surrounding South Orkney Islands region showed to be approximately 0.7°C higher in February 2017 compared with February 2016. This explains the one-month delay in sea ice formation observed in 2017, and thus the one-month extension in the ice-avoiding guild's acoustic presence and suggests that the distribution of these animals does follow the position of the ice edge. As the progressing sea ice made a higher proportion of the audible area inaccessible, and consequently reduced the prey availability due to inaccessibility, the animals were presumably pushed northwards and eventually out of the AURAL's audible area. Further support of the assumption of their migration back to northern breeding grounds comes from the appearance of humpback whale song-like vocalizations, which have been associated with migration and breeding behaviour, and the overall increase in fin whale vocal activity in the time period coinciding with the beginning of mating season [54,64,67,123].

As winter progressed, the observed shift in species composition reflects marine mammals' differential habitat preferences due to their contrasting ice-avoiding and ice-affiliated nature [20,64,124]. Minke whales are well adapted for a life within the pack ice and utilize the sea ice for both foraging and as habitat [75,125]. They have a robust rostrum which can be used to make breathing holes in the ice, and their relatively small and sleek body, and small flippers, allow them to move within the ice and get protection against predators [7,20,125]. Such adaptations enable them to utilize krill in areas with heavy sea ice cover, which is out of reach for most other species [126]. Bio-duck calls have been suggested to be associated with feeding activity, though evidence of this is lacking [55,63], and the detection of these calls during winter would coincide with their under-ice foraging strategy. Little is known about minke whales' distribution and migration patterns [76]. However, Shabangu *et al*. [55] stated that their presence is often associated with pack ice, and their close association with sea ice has been reflected in other studies [75,125]. In our study, minke whale acoustic presence showed a positive trend with increasing sea ice, and thus, these animals presumably moved into the audible area as the sea ice edge moved northwards and closer to the AURAL.

Minke whales are regarded as one of the largest ice-dependent krill predators in the SO. Thus, they may be especially vulnerable to changes in SIC and krill distribution [26,75,127]. Herr *et al*. [127] reported that minke whale distribution is strongly associated with the ice edge position and that a relatively low number of minke whales were spotted in areas with reduced winter sea ice duration. Though the current study only comprises two winter seasons, the apparent decrease in minke whale presence in relation to delayed ice formation in 2017 may serve as a preview for how future climate change can impact the distribution of these animals. This prolonged period of relatively ice-free water in 2017 may have led to a higher degree of interspecific competition for food due to the ice-avoiding guild extending their stay. As such, minke whales may have relocated further south to locate better ice conditions and prey availability. The observed response of minke whales and the ice-avoiding guild to the environmental anomalies observed in our study reflects that ice-affiliated species may be especially vulnerable to climate change and was further supported by the acoustic pattern observed for the two pinniped species.

Like minke whales, leopard and crabeater seal vocalizations mainly appeared in periods with heavy sea ice cover. However, the inter-annual difference in vocal activity between minke whales and the two pinnipeds was inverted, with pinniped vocalization being virtually absent during the El Niño year. Stuecker *et al*. [98] reported that from the summer season in 2015/2016 to summer 2016/2017, the largest decrease in summer sea ice extent was observed in the SO, and in our study inter-annual SIC comparisons revealed a sudden drop during spring 2016 (October–November) at the South Orkney Islands. Leopard and crabeater seals are closely associated with pack ice, used for hauling out, moulting and pupping [26,103], and their acoustic absence in 2016 demonstrates how spring sea ice variability may impact these ice-affiliated species and provides further evidence of environmental variability impacting marine mammal distribution. It is important to note that recordings in 2016 ended in late August, and we cannot say for sure whether these animals appeared later in spring. In 2017, both seal species were detected during austral spring/early summer, which coincides with their breeding season [103]. The observed positive association between the two seal species is a probable result of similar habitat preferences and a predator–prey relationship between the two. While leopard seals are generalists and eat a range of prey, such as krill, fish, penguins and other seals [103,128,129], crabeater seals are highly specialized foragers and are considered as one of the most prominent krill consumers in the SO [26]. Close association of krill with sea ice [130,131] may explain why crabeater seals are more confined to the pack ice than leopard seals, which have shown to disperse beyond the sea ice edge and spend noticeably more time in open water [103,132,133]. The generalized foraging nature of leopard seals also enables them to have a selective diet based on season and availability. As these animals have been shown to prey on crabeater seal pups, they may modulate their feeding behaviour and location in relation to crabeater seal pupping and weaning season [103,128,129].

### 4.2. Climate change and future research

The additional impact of extreme weather events such as ENSO has the potential to highly exacerbate the already strong seasonality characterizing polar regions and may result in significant inter-annual variation in environmental conditions [31–33]. The cascading effect following such variations in temperature, ocean circulation and subsequently sea ice extent impacts the entire food chain. Despite the Scotia Sea ecosystems being especially prone to both short- and long-term environmental changes due to its relation to the ACC and already strong seasonality, there are still a lot of unanswered questions regarding the impact of such alterations. Several studies have drawn parallels and links between large-scale distribution (e.g. seasonal migration) of marine mammals, prey availability and environmental changes [4,131,134–136]. However, information about how such patterns and relationships fluctuate at mesoscale (days and weeks) in more spatially restricted regions are scarce. Additionally, though we can observe inter-annual differences in the abundance and distribution of krill in restricted regions through the fisheries' catch data, little is known about the exact cause of krill biomass fluctuations and subsequently how such changes affect the marine mammals. The ability to predict inter-annual changes in regions within the Scotia Sea, which are characterized by ecosystem variability, is crucial due to the extensive overlap in resource exploitation between the commercial krill fishery and marine mammals [137,138], and especially due to future climate change.

Considering all the detected marine mammal species at South Orkney Islands in our study, coupled with visual sightings made during the Commission for the Conservation of Antarctic Marine Living Resources' (CCAMLR's) annual survey season [39,139], and the region being an important commercial krill fishing ground, the ecological and commercial importance of the South Orkney Islands is evident. Consequently, further monitoring and exploration of environmental and ecosystem variability is crucial for sustainable management of the marine resources and species residing in the area. By extending the current study by continuing the collection of year-round PAM data over multiple years in the future, we can gain novel information about long-term trends in the marine mammals' seasonal use of the island region, species co-occurrence and whether their acoustic phenology changes over time. Further, by combining it with more fine-scale monitoring of environmental changes and anomalies, we can see whether deviations or changes in acoustic presence coincide with a changing environment. Using multisensory tags, collecting movement patterns alongside acoustic data, in tandem with PAM could be used to explain potential deviations in acoustic presence by giving insight into whether the animals are moving to different feeding grounds, back to low-latitude breeding grounds, or if they are physically present but simply not vocalizing.

## 5. Conclusion

Our study clearly showed the importance of the South Orkney Islands as a feeding ground for a range of species, both migratory and residential, and documented temporal variation in both marine mammal species occurrence and assemblage. Additionally, it will increase our understanding of the seasonal presence of marine mammals at the South Orkney Islands, and how these animals may be affected by short- and long-term environmental changes. While the acoustic detections of ice-avoiding species reflected a prolonged stay at the South Orkney Islands in relation to the environmental anomalies, the ice-affiliated and ice-obligate species presented contrary inter-annual patterns. The observed inter-annual difference in marine mammal acoustic phenology in our study reflects the importance of collecting year-round data to gain an increased understanding of how environmental changes and extreme weather events modulate the movement strategies of marine mammals, as the variability may well fall outside the window where more traditional observation approaches such as vessel surveys take place. This is particularly relevant given the likelihood of increased frequency and intensity of rare weather events such as ENSO through continued climatic warming. The ability to better predict how marine mammals respond to a changing environment is crucial for advising CCAMLR, conservation decision processes and the fishery management.

## Data Availability

Acoustic spectrograms depicting marine mammal vocalizations can be found in the electronic supplementary material, accompanied by sound files for each spectrogram. Our data used in analyses pre- and post-review are deposited at 10.5281/zenodo.7670211 [140] and 10.5281/zenodo.8278598 [141], respectively. Supplementary material is available online [142].
